# LC–MS/MS quantification of olanzapine in hair after alkaline digestion

**DOI:** 10.1002/dta.3744

**Published:** 2024-06-05

**Authors:** Zeynep Turkmen, Zeynep Arslan, Merve Oka, Murat Yayla, Isil Bavunoglu

**Affiliations:** ^1^ Department of Science, Institute of Forensic Sciences and Legal Medicine Istanbul University‐Cerrahpaşa Istanbul Turkey; ^2^ Department of Internal Medicine, Cerrahpaşa Medical Faculty Istanbul University‐Cerrahpaşa Istanbul Turkey

**Keywords:** drug abuse, drug‐related crimes, forensic toxicology, second‐generation antipsychotics

## Abstract

Olanzapine (OLZ), a second‐generation antipsychotic drug, is effective in the treatment of acute psychosis, schizophrenia, agitation, bipolar mania, and other psychiatric problems. Antipsychotics are prescribed drugs, which lead the drug abuser to illegal methods of access. This behavior also demonstrates the association of OLZ with criminal involvement, commonly observed at forensic crime scenes. The acute toxicity and even death resulting from OLZ exposure have been highlighted in numerous studies. Therefore, developing analytical techniques to detect OLZ is essential for forensic toxicology. This study aimed to develop a specific and reliable LC–MS/MS method for OLZ detection and quantification in hair samples. The method was validated in terms of selectivity, linearity, limit of detection (LOD), limit of quantification (LOQ), trueness, precision, and uncertainty. The range of linearity was between 0.1–100 ng/mg, with LOD and LOQ values established at 0.036 ng/mg and 0.1 ng/mg, respectively. All validation results are within acceptable parameters. The validated method has been applied to authentic hair samples. The variation of OLZ concentrations in 12 hair segments (2 from Case 1 and 10 from Case 2) from two drug‐positive patients, ranging from 0.131 to 0.460 ng/mg, is presented in this study. Although several studies have been conducted to determine OLZ in hair samples using segmental analysis via hair solubilization, this study is the first to determine OLZ in hair samples after “digestion” with comparative parameters prior to chromatographic analysis.

## Graphical abstract

This graphical abstract illustrates every step of the laboratory work in this manuscript. Sample collection, segmentation, and alkaline digestion of the hair followed by SPE are crucial factors in the detection of OLZ by LC‐MS/MS.

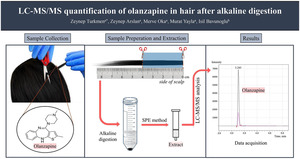

## 1 INTRODUCTION

Olanzapine (OLZ), a second‐generation atypical antipsychotic approved by the Food and Drug Administration (FDA) in 1996, is used primarily for the treatment of schizophrenia and bipolar disorder.^1^ OLZ is preferred for symptom improvement in schizophrenia patients because it has fewer extrapyramidal side effects than traditional antipsychotic drugs.^2^, ^3^ OLZ is generally associated with several adverse health effects, including weight gain, anticholinergic/extrapyramidal symptoms, cardiomyopathies, neurological abnormalities, sexual dysfunction, and increased all‐cause mortality.^4^ In addition to this, there are many cases in which acute toxicity from OLZ has occurred in adults, adolescents, and even infants, and has occasionally contributed to death.^5^, ^6^, ^7^ Furthermore, as OLZ is a prescription drug, abusers may resort to illegal means to obtain the drug. In some districts of the metropolitan city of Istanbul, pharmacies are robbed on a monthly basis. These substances regularly appear in court cases, especially in drug offenses.^8^, ^9^ OLZ, commonly known as “Lilly” among drug users, is taken orally, intravenously, or nasally to induce euphoria or sedation.^10^, ^11^ Although drug–drug interactions with OLZ are not prominent,^12^ most drug users combine OLZ with other substances at parties to induce pleasant euphoria (e.g., benzodiazepines, alcohol), reduce jitteriness and anxiety (e.g., cocaine), and enhance the effects of other illicit drugs.^13^ Considering all this information, the importance of OLZ in forensic toxicology is undeniable.

There are many studies in the literature on the detection and quantification of OLZ, particularly in blood and serum samples. The detection time of OLZ in blood varies due to its pharmacokinetic properties, making it more difficult to detect in postmortem cases due to its instability.^14^ Therefore, analytical methods as well as alternative biological samples (e.g., hair and nails) are needed to definitively detect and confirm the presence of OLZ. Hair samples offer advantages including ease of collection, longer preservation, and more accurate drug history.^15^ Segmental hair analysis can be used to confirm chronic use of a substance and also to determine the duration of use, as the growth rate is assumed to be approximately 1 cm/month.^16^ In this way, it can reveal a history of drug use, which can provide valuable information about the individual's drug use habits in any case. However, because hair contains very low concentrations, often in picograms per milliliter, sensitive analytical methods need to be used.

Liquid chromatography coupled with tandem mass spectrometry (LC–MS/MS) is an important tool in forensic toxicology for the identification and quantification of compounds in hair. Its sensitivity and selectivity make it effective even at low drug concentrations and with processed hair samples.^6^ However, rapid and reliable sample extraction and decontamination procedures are required to ensure accurate measurement. To date, organic drugs must be extracted by solubilization or digestion of the hair matrix itself, as there is no direct method for detecting organic drugs in the hair matrix.^17^ A few studies have attempted to determine OLZ concentrations in hair samples with or without segmental analysis by hair solubilization.^6^, ^18^, ^19^ However, this study represents the quantification of OLZ in hair after digestion following solid‐phase extraction (SPE) prior to chromatographic analysis. It is well known that the universal applicability of hair digestion is an efficient method for the release of all drug species from the protein matrix under certain conditions. Herein, we present for the first time the digestion procedure at different alkaline levels for the detection of OLZ. The developed method was successfully applied to authentic hair samples containing OLZ in each segment of the two drug‐positive patients.

## 2 MATERIALS AND METHODS

### 2.1 Chemicals and reagents

OLZ was purchased from Lipomed (Arlesheim, Switzerland), while cocaine‐d_3_ as an internal standard (IS) was purchased from LGC Standards (Middlesex, UK). Ammonium acetate (AmOAc), formic acid (FA), sodium hydroxide (NaOH), hydrochloric acid (HCl), LC‐grade methanol (MeOH), ethyl acetate (EA), and acetone (all of 99.8–100% purity) were purchased from Merck (Darmstadt, Germany). Welchrom BRP (60 mg, 3 mL) SPE cartridges were obtained from Welch Materials (Zhejiang, China). Minisart RC25 syringe filters (0.22 μm) were purchased from Sartorius (Göttingen, Germany). The experiments were conducted with nitrogen and argon gases of >99.999% purity purchased from Okser (Istanbul, Turkey). The process of nitrogen stream evaporation was conducted using the HyperVAP HV‐300 concentrator from Gyrozen (Daejeon, Republic of Korea). Direct‐Q 3 UV water purification system (18.2 MΩ cm) was acquired from Millipore (Molsheim, France). SPE negative pressure vacuum manifolds with 24 ports were used for the extraction of the samples (Phenomenex, USA).

### 2.2 Instrumental analysis

Analytical steps were carried out using a Shimadzu 8,045 LC device coupled with tandem MS (Kyoto, Japan). The positive ion mode was selected for electrospray ionization (ESI). Nitrogen gas was supplied from a Peak Scientific Genius 1051 device (Glasgow, Scotland). Chromatographic separation was performed on a Raptor Biphenyl column (2.7 μm, 100 × 2.1 mm) from Restek (Bellefonte, PA, USA) with mobile phases: (A) 2 mM AmOAc in 0.1% FA in ultrapure water and (B) 2 mM AmOAc in 0.1% FA in MeOH. The total run time was 17 min, with an injection volume of 2 μL. The column oven temperature was maintained at 50°C, while the interface temperature was maintained at 300°C. The flow rate was 0.4 mL/min. The gradient of mobile phase B was initiated at 5%, increased to 95% over 5 min, and then held for 4 min. For column equilibration. The dwell time was selected as 124.0 ms per transition to achieve maximum sensitivity. The heating gas flow and drying gas flow were set at 10 L/min. The analytes were detected by multiple reaction monitoring (MRM) with two transitions for each analyte: a quantifier and a qualifier. All analyses performed on the LC–MS/MS system were evaluated using Shimadzu's LabSolutions software.

### 2.3 Sample preparation

The stock solution of OLZ (analytical reference standard) was purchased as 1 mg/mL concentration in MeOH. Working (calibration) solutions containing the target substance were set to achieve the calibration curves. All calibration solutions, including 25 ng/mL of the IS, were incrementally prepared with 10 data points (0.1, 0.2, 0.5, 1, 2, 5, 10, 25, 50, and 100 ng/mg) in MeOH and blank human hair. A matrix‐matched calibration curve for the determination of OLZ was established using each calibration solution spiked with blank human hair to eliminate spectral interference.

Both drug‐positive and drug‐free hair samples provided voluntarily were used for calibration. Before use, the drug‐free hair (blank) sample was analyzed and confirmed to be free of OLZ. Any contaminants on the surface of the hair samples were removed by sequential washing with ultrapure water, acetone, and MeOH. The hair samples were finely cut into 1–2‐cm segments using sterile scissors and then dried at 25°C. Approximately 10 mg of each segmented hair was then weighed and cut into very small pieces. This increased the surface area of the hair and its interaction with NaOH. The crushed hair sample was transferred to a 50 mL tube to which 20 mL of 0.05 M NaOH was added. After optimization, all samples were analyzed accordingly. After overnight incubation at 50°C, the solution was neutralized to pH 10 using 1 M HCl solution. Before SPE, 25 ng/mL of IS was added and vortexed. The analytes were then extracted using an SPE column after preconditioning with the addition of 2 mL EA, 2 mL MeOH, and 2 mL ultrapure water. All samples were then loaded onto conditioned columns. After the loading step, the column was washed twice with 2 mL of 5% MeOH and then dried under 40 PSI nitrogen for 30 min. The analytes were eluted sequentially with 2 mL MeOH and 2 mL EA. The eluates were evaporated using nitrogen gas, and the residue was reconstituted in 1000 μL of MeOH. All samples were filtered before transfer to the vials. Finally, 2 μL of the samples was injected into an LC–MS/MS instrument with ESI in positive‐ion mode.

### 2.4 Method validation

The validation of the method was performed according to the Scientific Working Group for Forensic Toxicology (SWGTOX) guidelines for the limit of detection (LOD), the limit of quantification (LOQ), linearity, precision, selectivity, trueness, and stability.^20^ In addition, the uncertainty of the method was evaluated according to the EURACHEM CITAC and GUM documents.^21^, ^22^

Selectivity was evaluated by analyzing 10 different sources of blank human hair to identify chromatographic changes or interference from endogenous substances. To demonstrate linearity, the analytes were spiked with blank hair samples at known concentrations and analyzed using the optimized procedure. Linearity was confirmed by six replicates of each calibration point. Solvent and matrix‐matched calibrations were generated by adding known concentrations of analytes. Student's *t* test was used to compare the slopes of two calibrations. Peak area ratios of each analyte to IS were used to construct calibration curves. A correlation coefficient (*r*) > 0.99 was considered linear. The sensitivity of the proposed method is represented by the LOQ and LOD parameters. The LOD and LOQ values were obtained using the signal‐to‐noise (S/N) ratio estimated from 10 replicates of blank hair samples. The LOD and LOQ were calculated using S/N ratios of 3:1 and 10:1, respectively. The lowest concentration with acceptable trueness and precision was reported as the LOQ. Both intraday and interday evaluations were performed to evaluate trueness and precision. Six replicates for each of the three concentration levels (0.5, 5, and 50 ng/mg) were conducted on blank hair samples. Extraction efficiency was determined by computing the recovery percentage, which was used to establish trueness, while precision was assessed by estimating the relative standard deviation (RSD%) from six duplicate measurements. Both the extraction efficiency and RSD% calculations were based on the analyte responses observed in the blank hair sample. The assessment benchmarks were defined following the SWGTOX guidelines with a precision threshold of 20% RSD and a recovery range of 80–120%. To assess stability, the processed samples were measured in six replicates at 7‐day intervals. The calculated percentage change was considered acceptable if it remained below 1%, indicating stability. Uncertainty is derived from the evaluation of the validation results of the proposed method, which include purity of the standard sample (*u*_std_), calibration curve slope (*u*_cal_), recovery (*u*_rec_), and repeatability (*u*_rep_). The following equation was used to obtain the total uncertainty (*u*) resulting from the combination of all these factors:

$$u=\sqrt{{\left(u_{\mathrm{std}}\right)}^{2}+{\left(u_{\mathrm{cal}}\right)}^{2}+{\left(u_{\mathrm{rec}}\right)}^{2}+{\left(u_{\mathrm{rep}}\right)}^{2}}$$

To calculate the expanded uncertainty (*u*_exp_) at a 95% confidence level, multiply the combined uncertainty derived from the above equation by the coverage factor (*k* = 2). Table 1 summarizes the combined and expanded uncertainty results acquired during this validation.

**TABLE 1:** Results of combined and expanded uncertainty.

Compound	*u*_std_	*u*_cal_	*u*_rec_	*u*_rep_	*u*_com_	*u*_exp_
Olanzapine	0.98	1.01	0.03	0.02	1.40	2.81

*u*_std_: uncertainty of the standard sample, *u*_cal_: uncertainty of calibration curve slope, *u*_rec_: uncertainty of recovery, *u*_rep_: uncertainty of repeatability, *u*_com_: combined uncertainty, *u*_exp_: expanded uncertainty.

### 2.5 Collection of authentic samples

Hair samples were provided by the emergency department (ED) of Cerrahpaşa Medical Faculty to clarify the questioned cases. In order to determine the suspected use of OLZ, samples were taken by cutting hair from the vertex of the back of the head. The hair samples were placed in a 10‐mL screw‐capped glass tube and then transported to the Forensic Toxicology Laboratory at the Institute of Forensic Sciences and Legal Medicine of Istanbul University‐Cerrahpaşa. The samples were stored at room temperature until they were processed and analyzed for OLZ identification and quantification.

## 3 RESULTS

### 3.1 Method and sample preparation optimization

As the quantity of samples obtained in forensic cases is usually trace amounts, we aimed to use as little hair as possible in our study. Consequently, hair samples in varying amounts (10, 20, and 30 mg) were tested, with 10 mg proving to be sufficient and reliable for the detection of chronic OLZ use. Compared with larger amounts, 10 mg of hair was found to have a high recovery rate and a cleaner baseline. Therefore, 10 mg hair was chosen to avoid possible matrix effects in future analyses. Hair analysis is a complex process involving critical steps such as decontamination and extraction. The decontamination procedure used can have a significant effect on the analytical result of hair analysis.^23^ Therefore, attention should be paid to the efficacy of washing solutions, with the aim of removing as much as possible of the exogenous chemicals that accumulate on the outside of the hair, while preserving its content.

The most important part of hair digestion is the preservation of the analyte intact, depending on the chemical properties of the analyte.^24^ NaOH solution is one of the most effective and convenient ones for hair if the analyte(s) are stable under alkali conditions and can be effectively hydrolyzed by heating the hair sample.^25^, ^26^, ^27^ For compounds that are stable under alkaline conditions, digestion in an aqueous solution of NaOH followed by solvent extraction is an advantageous approach for the dissolution/extraction of the related compound from hair.^25^ Therefore, NaOH was chosen due to its efficiency in minimizing matrix effects, particularly in the detection of weakly basic drugs such as OLZ. Several incubation solvents including 0.05 M, 0.1 M, 1 M, and 20 M NaOH were also investigated for pretreatment of hair samples. After determining the incubation solvent, tests were conducted to investigate the effect of changing the pH during pretreatment, as well as varying the heating temperature (40°C, 50°C, and 65°C) and incubation time (120 min and overnight). The degradation of OLZ increased as the molarity of NaOH increased from 0.05 to 20 M (overnight incubation at 50°C), as shown in Figure S1, while changes in heating had no effect within the same pH range. The best recovery results were obtained for both types of sample preparation up to overnight incubation at 50°C with 0.05 M NaOH (Table S1). It is known that the hair matrix contains a variety of contaminants such as cosmetics and that drug concentrations in hair are typically very low. Therefore, to improve the recovery efficiency of our target drug and to remove contaminants, a further preconcentration process using SPE or liquid–liquid extraction (LLE) is necessary. SPE was preferred in our study because it requires less matrix effect, labor, and solvent than LLE. After optimal digestion and extraction procedures were set, OLZ recovery studies were conducted at three concentration levels at 0.5, 5, and 50 ng/mg with six replicates. Consequently, the digestion, extraction, and SPE methods actively used in the laboratory to extract illicit substances were improved to obtain a more noise‐free chromatogram.^28^, ^29^ In this context, good digestion performance and extraction efficiency for the detection and quantification of OLZ from hair were the focus of our study.

### 3.2 Method validation

**FIGURE 1:**
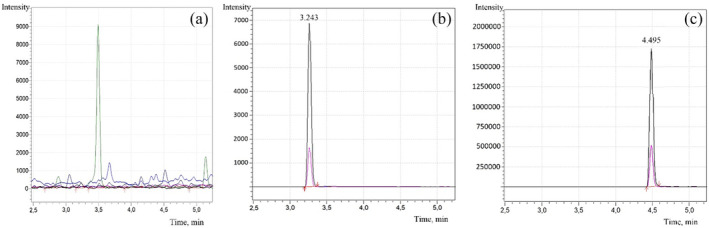
Chromatograms of samples with concentrations corresponding to blank samples (a), blank with 0.5 ng/mg olanzapine (b), and blank with cocaine‐d_3_ (IS) (c).

A quantitative LC–MS/MS method was developed for the detection and quantification of OLZ in human hair with this study. The chromatographic separation was efficiently achieved within 6.5 min, with no interference peaks originating from endogenous substances during analyte elution (Figure 1). The method exhibited selectivity for OLZ detection in human hair. Additionally, despite injecting the highest calibration standard, no significant carryover effect was observed. The quantification approach followed the SWGTOX guideline, utilizing the most prominent MRM transitions for quantification and a secondary transition for qualification. The parameters of tandem mass spectrometric detection are summarized in Table 2. Once optimized, the methodology was fully validated in spiked human hair and MeOH matrices. During method optimization, different mobile phases were explored to enhance resolution, selectivity, and peak sharpness. The slopes of calibration curves were similar in both matrices, as demonstrated by Student's *t* test (*p* > 0.05). As a result, matrix‐matched calibration in hair was used to avoid potential matrix effects in future investigations.

**TABLE 2:** Parameters of tandem mass spectrometric detection.

Compound	RT (minutes ± SD)	Q1 pre bias (V)	Collision energy (V)	Q3 pre bias (V)	MRM transition (m/z)
Olanzapine	3.243 ± 0.005	−30.0	−24.0	−27.0	313.1 > 256.1
		−11.0	−32.0	−14.0	313.1 > 213.1
Cocaine‐d_3_ (IS)	4.495 ± 0.001	−12.0	−21.0	−19.0	307.2 > 185.2
		−12.0	−31.0	−15.0	307.2 > 85.0

Abbreviations: IS, internal standard; RT, retention time; SD, standard deviation; MRM, multiple reaction monitoring.

Linearity was achieved within the range of 0.1–100 ng/mg, with an *R* value of 0.9994 in the human hair matrix. The regression equation of the calibration curve for OLZ was *y* = 0.0307*x*−0.0107. The LOD and LOQ values were 0.036 ng/mg and 0.1 ng/mg, respectively (Table 3). The developed method showed good trueness and precision at low, medium, and high concentrations (0.5, 5, and 50 ng/mg). Overall, the trueness values were within the 81.80–96.46% range. The RSD values of the intraday assay varied from 1.76 to 6.55%, while the interday assay outcomes varied from 2.12 to 8.95%. Therefore, the method's trueness and precision were both deemed satisfactory. The LOD, LOQ, trueness, and intraday and interday precision values are summarized in Table 3. The analyzed samples showed a change of 0.0054%, affirming the stability of the developed method. The mean matrix effect values ranged from 83.3 to 99.9% in six replicates of an analyte containing 1 ng/mg OLZ in human hair, with the coefficient variation (CV%) indicating no significant variation for OLZ. The calculated average matrix effect and process efficiency of an analyte containing 1 ng/mg OLZ in human hair were 91.55% and 77.01%, respectively. There were no changes in the matrix effect, recovery, and process efficiency across different concentration levels. All parameters fulfilled the acceptance criteria aligned with the SWGTOX guidelines. Also, the uncertainty values were determined to be appropriate based on the EURACHEM CITAC and GUM documents.

**TABLE 3:** Performance of the method by several parameters.

Compound	LOD (ng/mg)	LOQ (ng/mg)	Conc (ng/mg)	Rec (%)	Intra‐day assay (*n* = 6)			Inter‐day assay (*n* = 6)		
					Mean	±SD	RSD (%)	Mean	±SD	RSD (%)
Olanzapine	0.036	0.1	0.5	82.43	0.41	0.02	6.55	0.39	0.03	8.95
			5	81.80	4.09	0.15	3.67	3.95	0.27	6.83
			50	96.46	48.23	0.85	1.76	47.55	1.01	2.12

Abbreviations: Conc, concentration; LOD, limit of detection; LOQ, limit of quantification; Rec, recovery; RSD, relative standard deviation; SD, standard deviation.

### 3.3 Application to authentic samples

The validated method was applied to authentic hair samples obtained from ED. OLZ was detected in two cases, with the quantitative results summarized in Table 4. All samples were segmented and numbered from proximal to distal at 1‐cm intervals.

**TABLE 4:** Results of the total hair analysis for drug‐positive patients.

Patient no.	Gender	Hair length (cm)	Segment (cm)	Olanzapine concentration (ng/mg)
1	Female	2	0–1	0.224
			1–2	0.210
2	Female	10.5	0–1	0.288
			1–2	0.217
			2–3	0.160
			3–4	0.153
			4–5	0.139
			5–6	0.131
			6–7	0.354
			7–8	0.408
			8–9	0.460
			9–10.5	< LOQ

Abbreviations: < LOQ, the result is below the limit of quantification.

## 4 DISCUSSION

Numerous reports have highlighted cases of acute OLZ toxicity in adults and adolescents, even resulting in death.^5^, ^6^ While OLZ has been implicated in toxicity in some overdose scenarios, concrete evidence of this association remains limited. However, most deaths associated with OLZ are associated with multiple concomitant drug use and other physical risk factors.^14^ In addition, there appears to be a risk of abuse among certain drug users who are aware of the sedative and other pharmacological properties of OLZ. According to one case study, OLZ is used to enhance or substitute the effects of illicit drugs. Specifically, in combination with benzodiazepines and alcohol, OLZ has been observed to induce euphoria and relieve the tension and anxiety produced by cocaine.^13^ The actual number of potential cases of drug‐related toxicity is likely to be higher. This is due to difficulties in accessing data; in many cases, forensic toxicology plays an important role in deaths caused by OLZ abuse, whether intentional or accidental, including suicides.

There are many studies dealing with methods for the determination of OLZ in hair, as it is of great importance in forensic and clinical toxicology. The analytical determination of OLZ, particularly in blood and serum samples, has been a topic of discussion in scientific publications for over a decade, with ongoing method refinements.^14^ In this context, alternative samples (such as hair and nails) complement traditional samples (such as blood and urine), and methods to analyze these samples accurately are needed to determine the presence of OLZ. Along with this, as it is known, the digestion stage is very important in the liquefaction of solid biological samples such as hair and nails before extraction. The most important part of digestion is to preserve the analyte intact depending on its chemical properties.

In the literature review on digestion conditions, it was found that some major drug groups such as antipsychotics, antidepressants, and amphetamines were frequently reported to be extracted by alkaline digestion (especially NaOH).^30^, ^31^, ^32^, ^33^ It is also more advantageous for the detection of cannabinoids.^33^ In contrast, NaOH digestion has some disadvantages in the determination of opiates due to the decomposition of substances such as acetyl‐morphine and acetyl‐codeine into morphine and codeine.^34^ Similar to our study, NaOH at concentrations ranging from 0.5 to 2.5 M was used to digest the hair sample to determine the content of polycyclic aromatic hydrocarbons, nicotine, and cotinine in hair samples.^35^ In addition, several studies have reported mechanical effects such as ultrasound assistance and/or heating to easily hydrolyze creatine with NaOH.^27^ On the other hand, in our unpublished study, we found that 1 M NaOH was sufficient for the alkaline environment to digest the nail, which is a more keratinized structure than hair, for the detection of nicotine and cotinine.^36^ In light of the above information, the chemical structure of the target molecule should not be ignored when performing digestion and extraction.

Due to its high selectivity, and specificity, the LC–MS/MS method is effectively applied to hair after the isolation of the accumulated drug, even at low drug concentrations and/or with processed hair samples.^6^ To the best of our knowledge, there is limited data in the literature related to OLZ detection in hair samples by LC–MS/MS. A study akin to ours has focused on establishing reference values for OLZ and *N*‐desmethyl‐OLZ concentrations in postmortem hair from chronic OLZ users, contributing to the establishment of a reference range for this drug.^6^ Günther et al., in their study, worked with 37 postmortem hair samples, and the linear range was stated as 0.005–10.00 ng/mg for OLZ and 0.025–5.00 ng/mg for *N*‐desmethyl‐OLZ. The average extraction recovery in the study using the ACQUITY UPLC HSS C18 column (1.8 μm, 150 × 2.1 mm) was 79% for OLZ and 60% for the metabolite. Furthermore, the same study emphasized the crucial role of method selection in OLZ determination.^6^ Wang et al., in their study with Allure PFP propyl column (5 μm, 100 × 2.1 mm), worked with five schizophrenic patients in the linear range of 0.05–20 ng/mg, with an *R* value of 0.9935, for OLZ in hair samples, and recovery was above 79%. Also, the optimized method for extraction reported by Wang et al. did not offer high reproducible extraction yields for OLZ.^18^ Cobo‐Golpe et al. used the SPE method like ours in their study with the XBridge BEH Shield RP18 (2.1 × 5 mm, 3.5 m) guard column in 2020. They worked with five patients in the linear range of 10–10,000 pg/mg for OLZ in nail and hair samples. For hair samples, the extraction recovery ranged from 45.1 to 83.6%, while for nail samples, it ranged from 62.3 to 109.8%.^19^ Given its heightened sensitivity, as evidenced by an *R* value of 0.999, the LC–MS/MS method with Raptor Biphenyl column (2.7 μm, 100 × 2.1 mm) employed in our study establishes a linear range across 10 data points, demonstrating its ability to perform a wide range of quantitative analyses. In the current study, the validated method has some advantages over other available methods, including less sample volume (10 mg), a short analysis timeframe (6.5 min), a low injection volume (2 μL), and high recovery values. This is important because usually the digestion and extraction processes not only require large amounts of hair but also have hard labor issues in preparation of the sample and consume a lot of reagents.

Once we had validated the developed method, we verified it on authentic samples, as it reflects the basic approach in all forensic toxicology investigations. Although the number of cases was limited, the validated method was successfully applied to authentic samples in order to clear doubt. Furthermore, it demonstrated its capability to successfully detect OLZ in hair samples spanning a length of 10.5 cm. Analytical results can be strongly influenced by factors such as the amount of substance consumed, drug metabolism, the position of the drug along the hair (proximal or distal), hair color, and polarity of the target drug.^37^ Therefore, it was confirmed that both patients in our study had dark brown hair and that their hair was not dyed, and we aimed to eliminate other potential factors that could alter the hair concentration as well as the dosage. Further studies are needed to ascertain a potential correlation between hair color, dosage, and hair concentration. OLZ was detected in the hair samples of two cases, and the data obtained for each 1‐cm segment group are given in Table 4. The total of 12 segments (2 from Case 1 and 10 from Case 2) include concentrations ranging from 0.131 to 0.460 ng/mg. It is known that drug concentrations generally decrease from the proximal to the distal end of the hair. Therefore, one would expect lower OLZ concentrations in the distal segments of individuals with a history of chronic use. However, contrary to the literature, our study showed that the overall OLZ concentration in Case 2 increased from proximal to distal segments. The variation in drug levels in each hair segment in Case 2 indicated that she had been using OLZ for approximately 8–9 months and that the amount ingested had recently decreased compared with baseline. A decrease in concentration may indicate reduced or discontinued OLZ use.

Although several studies have been conducted to determine OLZ in hair samples via hair solubilization, this study represents the first determination of OLZ in hair after alkaline digestion prior to chromatographic analysis. Our study was improved for the detection and quantification of OLZ using a combination of alkaline digestion and advanced techniques such as SPE, ESI, and MS/MS. In this manner, this study provided a simple and reliable method with good extraction efficiency and digestion performance for the detection and quantification of OLZ in hair samples, which is very important in criminal investigation. The determination of OLZ in the hair matrix is essential for the confirmation of drug‐related intoxications or deaths as well as for abuse potential. Therefore, future research in this area should include the development of substance‐specific extraction methods that do not degrade the drug structure when extracted from biological matrices such as nails and vitreous humor.

## 5 CONCLUSION

This study was conducted to develop a specific and reliable method for the quantification of OLZ in hair samples using the LC–MS/MS method with a combination of alkaline digestion and SPE. Using this validated method, suspected OLZ use can be monitored by analyzing each hair segment. It provides a reliable scientific basis for toxicological analysis in forensic cases such as poisoning or drug abuse. Therefore, the impact of this study will be a significant improvement in the ability to solve cases of chronic use of OLZ. This study demonstrates that the OLZ drug substance in hair is suitable for measurement after alkaline digestion, which is known to be universally applicable, and has sufficient sensitivity and selectivity to overcome the problems of matrix components that may cause interference.

## CONFLICT OF INTEREST STATEMENT

The authors declare no conflicts of interest.

## Supporting information


**Table S1.** Optimization performance results.

**Figure S1.** Recovery values of olanzapine at various NaOH molarity
