# Supplementary material for: LC–MS/MS quantification of olanzapine in hair after alkaline digestion
Source: Drug Test Anal. 2024 Jun 5;17(3):412–9. doi: 10.1002/dta.3744 (PMC11922687; doi:10.1002/dta.3744)
Supplement: Supplementary file 1 — Table S1. Optimization performance results. [file DTA-17-412-s002.docx]

**Table S1**. Optimization performance results.

|  |  | **Recovery of Olanzapine (%)** |
| --- | --- | --- |
| **NaOH Molarity** | 0.05M | 85.32 |
|  | 0.1M | 84.78 |
|  | 1M | 18.92 |
|  | 20M | 6.78 |
| **Temperature degree** | 40°C | 82.38 |
|  | 50°C | 85.32 |
|  | 65°C | 85.41 |
| **Incubation duration** | 120 minutes | 85.32 |
|  | Overnight | 94.76 |
