# Supplementary figures and images for: LC–MS/MS quantification of olanzapine in hair after alkaline digestion
Source: Drug Test Anal. 2024 Jun 5;17(3):412–9. doi: 10.1002/dta.3744 (PMC11922687; doi:10.1002/dta.3744)

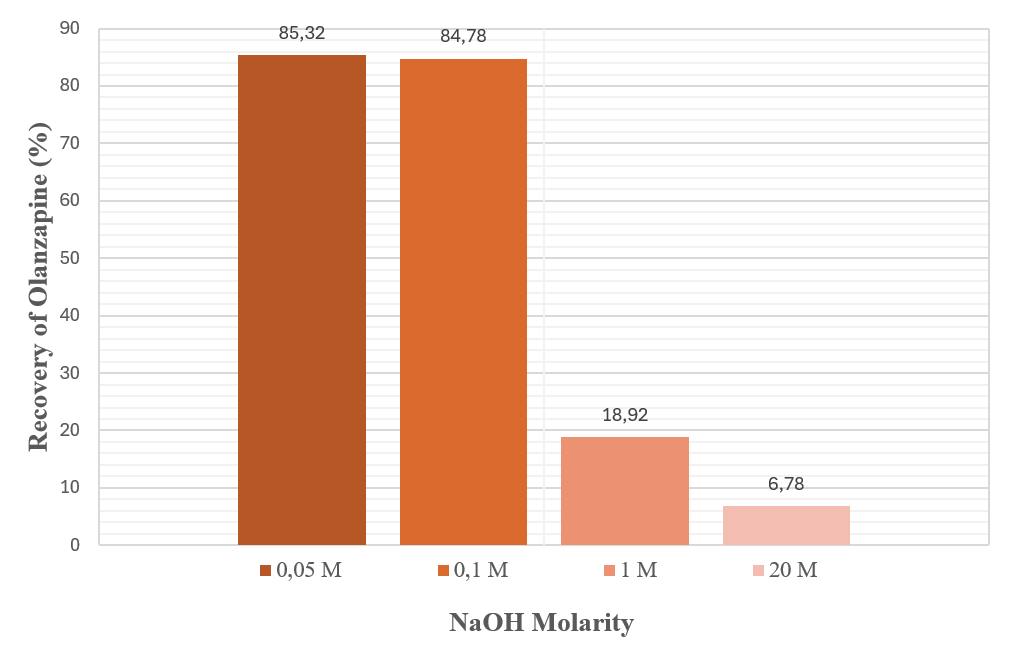

Supplement: Supplementary file 2 — Figure S1. Recovery values of olanzapine at various NaOH molarity [file DTA-17-412-s001.png]
